# Hf(OTf)_4_ as a Highly Potent Catalyst for the Synthesis of Mannich Bases under Solvent-Free Conditions

**DOI:** 10.3390/molecules25020388

**Published:** 2020-01-17

**Authors:** Shuai-Bo Han, Jing-Ying Wei, Xiao-Chong Peng, Rong Liu, Shan-Shan Gong, Qi Sun

**Affiliations:** Jiangxi Key Laboratory of Organic Chemistry, Jiangxi Science and Technology Normal University, 605 Fenglin Avenue, Nanchang 330013, China

**Keywords:** hafnium triflate, Mannich reaction, solvent-free, β-amino carbonyl compound, mechanism

## Abstract

Hf(OTf)_4_ was identified as a highly potent catalyst (0.1–0.5 mol%) for three-component Mannich reaction under solvent-free conditions. Hf(OTf)_4_-catalyzed Mannich reaction exhibited excellent regioselectivity and diastereoselectivity when alkyl ketones were employed as substrates. ^1^H NMR tracing of the H/D exchange reaction of ketones in MeOH-*d*_4_ indicated that Hf(OTf)_4_ could significantly promote the keto-enol tautomerization, thereby contributing to the acceleration of reaction rate.

## 1. Introduction

Mannich reaction has been recognized as one of the most classic multicomponent reactions (MCRs) and utilized for the synthesis of β-amino carbonyl compounds (Mannich bases) via one-pot condensation-addition of aldehyde, amine, and ketone since its discovery in 1917 [1]. Mannich bases are versatile synthetic intermediates [2,3,4,5] and widely applied in the synthesis of natural products [6] and pharmaceutical chemistry [7,8].

In past two decades, Brønsted acid-based catalysts, such as conc. HCl [9], camphor sulfonic acid [10], HClO_4_-SiO_2_ [11], polymer-supported sulfonic acid [12], H_3_PW_12_O_40_ [13], acidic surfactants [14], and acidic ionic liquids [15], have been extensively explored for Mannich reaction, which provides a reliable access to Mannich bases. However, these methods are typically limited by large catalyst loading, moderate yield and long reaction time. Meanwhile, various metal Lewis acids, such as Yb(OPf)_3_ [16], ZrOCl_2_·8H_2_O [17], Zn(OTf)_2_ [18], NbCl_5_ [19], SnCl_2_/SnCl_4_ [20,21], BiCl_3_ [22], CeCl_3_·7H_2_O/CAN [23,24], FeCp_2_PF_6_ [25], and Ga(OTf)_3_ [26] have been employed for the synthesis of Mannich bases under either solution-phase or solvent-free conditions. In addition, organometallic complexes of Ti(IV) [27], Bi(III) [28], Sb(III) [29], Zr(IV) [30,31], along with other Lewis acids, such as sulfonium [32]/iodonium salts [33] and SiCl_4_ [34], have also been proved as effective catalysts for this purpose. However, these methods typically require at least 5–10 mol% catalyst. Therefore, highly potent, low-cost, and non-toxic metal Lewis acid catalysts for Mannich reaction are still highly desired. Recently, novel heterogeneous catalysts such as nanoparticle-supported/encapsulated solid acids [35,36,37], mesoporous materials [38,39], metal nanoparticles [40], and metal-coordinated polymers [41,42] have provided improved catalyst recyclability. However, the preparation of these specific catalysts greatly limits their practical applications.

Previous research on Group IVB transition metal (Zr(IV) and Hf(IV)) catalysts revealed their superior activity on many carbonyl-transformation reactions [30,43]. Our ongoing research in this field showed that Hf(IV) salts are even more potent than Zr(IV) salts in many carbonyl-transformation reactions [44,45,46]. However, the catalytic effect of Hf(IV) salts on Mannich reaction has never been explored. We report herein the identification of Hf(OTf)_4_ as a highly potent catalyst for efficient synthesis of a diversity of β-amino carbonyl compounds under solvent-free conditions at room temperature. The alkyl ketone-based Mannich reaction catalyzed by Hf(OTf)_4_ exhibited excellent regioselectivity and diastereoselectivity. The H/D exchange experiments showed that Hf(OTf)_4_ catalyst could significantly promote the rate of keto-enol tautomerization.

## 2. Results and Discussion

### 2.1. Aryl Ketone-Based Mannich Reaction Catalyzed by Hf(OTf)_4_

In the preliminary experiment, the catalytic activity of Zr(IV) and Hf(IV) salts at 5 mol% level was compared in a model reaction, which contained benzaldehyde, aniline, and acetophenone in a 1:1:2 molar ratio at room temperature. The results in Table 1 showed that the aryl ketone-based Mannich reaction could not proceed without catalyst. The catalytic activity of ZrCl_4_, ZrOCl_2_·8H_2_O, and ZrCp_2_Cl_2_ were close (~70% yield, 24 h). In contrast, HfCl_4_ (81%, 8 h) and Hf(OTf)_4_ (89%, 6 h) exhibited remarkably higher activity than Zr(IV) salts.

The solvent effect was investigated in the presence of 5 mol% Hf(OTf)_4_. As listed in Table 2 (entry 1–4), the reactions in THF, DME, benzene, and CH_2_Cl_2_ proceeded much slower with good to moderate yields. Compared to the reaction in acetonitrile, the one performed in EtOH resulted in shorter reaction time and higher yield (94% yield, 5 h). But when the reaction in EtOH was elevated to 80 °C, to our surprise, the reaction rate and yield of Hf(OTf)_4_-catalyzed Mannich reaction was not significantly affected like many other reactions [42,43,44].

As expected, reducing the amount of Hf(OTf)_4_ catalyst to 2 mol% for the reaction in EtOH resulted in prolonged reaction time (12 h). In contrast, under solvent-free conditions, 2 mol% Hf(OTf)_4_ furnished efficient formation of **1** in only 2 h at room temperature. Further optimization determined that the amount of Hf(OTf)_4_ could be reduced to as low as 0.5 mol% for efficient production of **1** (93%, 4 h).

**Scheme 1:**
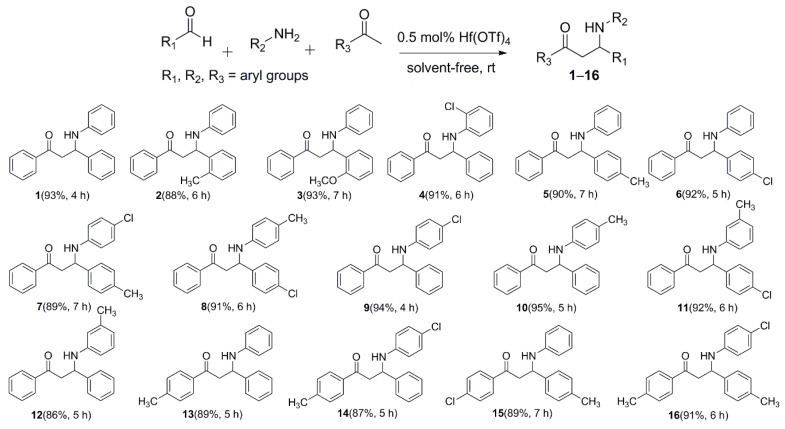
Hf(OTf)_4_-catalyzed synthesis of aryl ketone-derived Mannich bases **1**–**16**. Aldehyde/aniline/aryl ketone are in a 1:1:2 molar ratio.

With the optimized conditions, 0.5 mol% Hf(OTf)_4_ was applied to the synthesis of a diversity of aryl ketone-derived β-amino carbonyl compounds (**1**–**16**). As shown in Scheme 1, the current method exhibits good generality to a variety of substrates, and the aryl ketone-derived Mannich bases were obtained in excellent yields (87–94%) within 4–7 h. Electron-donating and electron-withdrawing substituents at the *ortho*, *meta*, and *para* positions of the phenyl rings of ketone, aldehyde, and aniline are well tolerated by this method.

### 2.2. Alkyl Ketone-Based Mannich Reaction Catalyzed by Hf(OTf)_4_: Regioselectivity and Diastereoselectivity

Since the reactivity of alkyl ketones are typically higher than that of aryl ketones, the reaction of benzaldehyde, aniline, and acetone (2:1:1 molar ratio) only took 10 min in the presence of 0.5 mol% Hf(OTf)_4_ under solvent-free conditions. Further optimization determined that only 0.1 mol% Hf(OTf)_4_ is sufficient to catalyze the high-yielding formation of the Mannich base **17** (94%, 30 min).

**Scheme 2:**
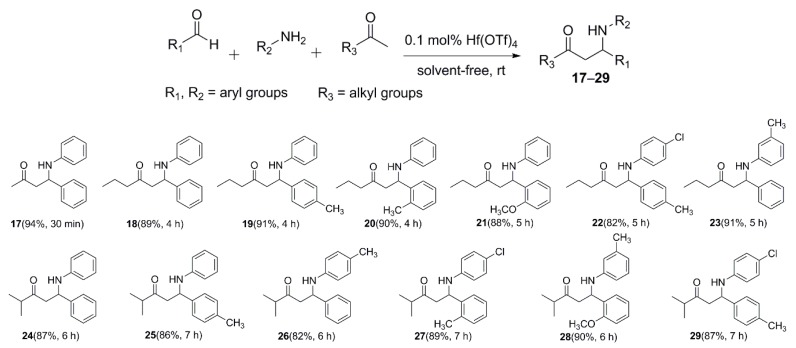
Hf(OTf)_4_-catalyzed synthesis of alkylketone-derived Mannich bases **17**–**29**. Aldehyde/aniline/alkyl ketone are in a 1:1:2 molar ratio.

However, when more complicated alkyl ketones such as 2-pentanone and 1,1-dimethylacetone were used, the reaction rate was notably slower. It took 4–5 h to yield the corresponding Mannich bases **18** and **24** in good yields. More importantly, comparison with the reactions without catalyst indicated that the presence of Hf(OTf)_4_ not only promoted the reaction rate but also resulted in high regioselectivity. As shown in Table 3, in contrast to the uncatalyzed reactions, which yielded both isomers (**a**:**b** = ~1:0.5 molar ratio, determined by ^1^H NMR), only the less substituted, namely the methyl-derived, isomer (**a**) was obtained when Hf(OTf)_4_ was used. The application of the optimized conditions to linear alkyl ketone substrates afforded a diversity of Mannich bases **18**–**29** in excellent yields (82–91%) within 4–7 h (Scheme 2).

In the following research, we investigated the diastereoselectivity of Hf(OTf)_4_-catalyzed synthesis of cycloketone-derived Mannich bases **30**–**32** under solvent-free conditions. We noticed that even residual Hf(IV) cation in the used round-bottom flask may significantly affect the outcome of the *anti*/*syn* ratio. To avoid false results from the contamination of the trace amount of residual catalyst, the control reactions without catalyst were all performed in new reaction vessels with new stir bars. In addition, the ratio of *anti*/*syn* isomers was determined directly from ^1^H NMR of the crude product. The results listed in Table 4 showed that the Mannich reaction of cyclopentanone did not yield the desired product at all after 12 h without catalyst. For cyclohexanone and cycloheptanone, the corresponding reactions were sluggish and poor yielding. When 0.1 mol% Hf(OTf)_4_ was applied, both the reaction rates and yields of these reactions were notably improved. The presence of Hf(IV) cation dramatically increased the ratio of *anti*/*syn* isomers from 63:37 up to 96:4 when cyclohexanone was used. For cyclopentanone, Hf(IV) cation also favored the formation of *anti* isomer (*anti*/*syn* = 92:8). But increasing the amount of Hf(OTf)_4_ to 1 mol% did not further improve the ratio of *anti*/*syn* isomers. Interestingly, in the case of cycloheptanone, solvent-free conditions alone favored the formation of *syn* isomer, but addition of 0.1 mol% Hf(OTf)_4_ increased the ratio of *anti*/*syn* isomers from originally 20:80 to 59:41. It was observed that higher Hf(OTf)_4_ loading resulted in remarkable increase in both reaction rate and *anti*/*syn* ratio, which could reach up to *anti*/*syn* = 86:14 when 50 mol% Hf(OTf)_4_ was used.

### 2.3. The Catalytic Role of Hf(OTf)_4_ on Keto-Enol Tautomerization

**Figure 1:**
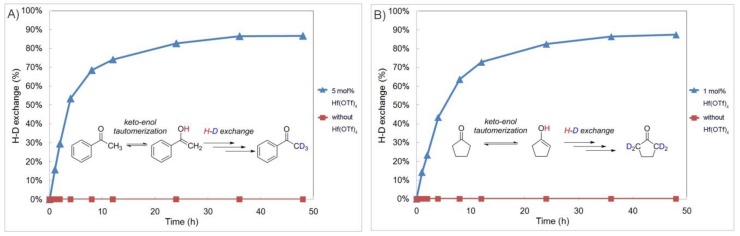
The H/D exchange reactions of acetophenone (**A**) and cyclopentanone (**B**) in MeOH-*d*_4_ in the absence or presence of Hf(OTf)_4_.

In our previous research, we have revealed Hf(IV) cation’s strong capability on activating benzaldehyde for the fast formation of the imine intermediate [45]. Many previous reports had also proposed that the interactions of metal cations with ketone are also involved in the catalysis of Mannich reaction. However, not much evidence has been provided to support this point. In the current research, we utilized ^1^H NMR to examine the activation effects of Hf(IV) on both aryl ketone and alkyl ketone. Interestingly, when 5 mol% Hf(OTf)_4_ was added to acetophenone in MeOH-*d*_4_, a remarkable H/D exchange process was promoted. As shown in Figure 1A, 86.6% of the proton of the active methyl group was exchanged to deuterium over 36 h. For cyclopentanone, only 1 mol% Hf(OTf)_4_ was needed to result in a comparable H/D exchange process (87.5%, 36 h), which is in agreement with the result that less catalyst is required for alkyl ketone-based Mannich reaction. Similar to the promoted tautomerization of dimethylphosphite in Kabachnik reaction [45], the coordination of Hf(IV) dramatically accelerated the tautomerization between the ketone and enol forms of both aryl and alkyl ketone, thereby increasing the overall reaction rate.

## 3. Materials and Methods

### 3.1. General Methods

General chemical reagents and solvents were obtained from commercial suppliers. All reactions were monitored by thin layer chromatography on plates coated with 0.25 mm silica gel 60 F254 (Qingdao Haiyang Chemicals, Qingdao, China). TLC plates were visualized by UV irradiation (254 nm, Shanghai Peiqing Sci & Tech, Shanghai, China). Flash column chromatography employed silica gel (particle size 32–63 μm, Qingdao Haiyang Chemicals, Qingdao, China). Melting points were determined with a Thomas-Hoover melting point apparatus (Thomas Scientific, Swedesboro, NJ, USA) and uncorrected. NMR spectra were obtained with a Bruker AV-400 instrument (Bruker BioSpin, Faellanden, Switzerland) with chemical shifts reported in parts per million (ppm, δ) and referenced to CDCl_3_ or DMSO-*d*_6_. The NMR spectra of compounds **11**, **15**, **16**, **20**–**23**, **25**, and **27**–**29** were provided in Supplementary Materials (Figures S1–S22). IR spectra were recorded on a Bruker Vertex-70 spectrometer (Bruker Optics, Billerica, MA, USA). High-resolution mass spectra were reported as *m*/*z* and obtained with a Dalton micrOTOF-Q II spectrometer (Bruker Daltonics, Billerica, MA, USA).

### 3.2. General Synthetic Procedure and Characterization of Mannich Bases

To a mixture of aldehyde (2.0 mmol), aniline (2.0 mmol), and ketone (4.0 mmol) was added Hf(OTf)_4_ (10 μmol for aryl ketones or 2 μmol for alkyl ketones). The reaction was stirred at room temperature (0.5–7 h) and monitored by TLC (Jone’s reagent as stain for Mannich base). Upon completion, CH_2_Cl_2_ (50 mL) was added to the reaction to dissolve the residue. The organic phase was washed with NaHCO_3_ solution (50 mL), dried over anhydrous Na_2_SO_4_, and concentrated in vacuo. Flash column chromatography (hexane/ethyl acetate 10:1) afforded the desired Mannich base in pure form. The characterization data of Mannich bases **1**–**10**, **12**–**14**, **17**–**19**, **24**, **26**, and **30**–**32** were in good agreement with those reported in literatures [17,19,20,22,25,36,47,48,49,50].

*3-(4-Chlorophenyl)-1-phenyl-3-(m-tolylamino)propan-1-one* (**11**): a white solid; mp 109–110 °C. ^1^H NMR (400 MHz, CDCl_3_): δ 7.91 (d, *J* = 7.6 Hz, 2H), 7.58 (t, *J* = 7.4 Hz, 1H), 7.46 (dd, *J*_1_ = 8.1 Hz, *J*_2_ = 7.6 Hz, 2H), 7.39 (d, *J* = 8.3 Hz, 2H), 7.29 (d, *J* = 8.4 Hz, 2H), 7.00 (t, *J* = 7.8 Hz, 1H), 6.51 (d, *J* = 7.4 Hz, 1H), 6.40 (s, 1H), 6.33 (d, *J* = 8.0 Hz, 1H), 4.99 (dd, *J*_1_ = *J*_2_ = 6.2 Hz, 1H), 4.50 (br, 1H), 3.47 (dd, *J*_1_ = 5.4 Hz, *J*_2_ = 16.2 Hz, 1H), 3.41 (dd, *J*_1_ = 7.3 Hz, *J*_2_ = 16.3 Hz, 1H), 2.21 (s, 3H) ppm; ^13^C NMR (100 MHz, CDCl_3_): δ 198.1, 146.9, 141.8, 139.1, 136.9, 133.7, 133.1, 129.2, 129.1 (×2), 128.9 (×2), 128.4 (×2), 128.0 (×2), 119.2, 115.0, 111.0, 54.4, 46.3, 21.7 ppm; IR (KBr) *ν*_max_ 3389, 3063, 2918, 1676, 1602, 1593, 1519, 1489, 1447, 1411, 1372, 1329, 1305, 1288, 1257, 1217, 1180, 1089, 1066, 1014, 1001, 990, 840, 826, 776, 756, 722 cm^−1^; HRMS (ESI+): *m*/*z* calcd for C_22_H_22_ClNO [M + H]^+^ 350.1306; found 350.1309.

*1-(4-Chlorophenyl)-3-(phenylamino)-3-(p-tolyl)propan-1-one* (**15**): a white solid; mp 121–122 °C. ^1^H NMR (400 MHz, CDCl_3_): δ 7.84 (d, *J* = 8.6 Hz, 2H), 7.41 (d, *J* = 8.6 Hz, 2H), 7.32 (d, *J* = 8.0 Hz, 2H), 7.11 (dd, *J*_1_ = *J*_2_ = 16.6 Hz, 1H), 6.68 (t, *J* = 7.3 Hz, 1H), 6.57 (d, *J* = 7.8 Hz, 2H), 4.98 (dd, *J*_1_ = *J*_2_ = 6.3 Hz, 1H), 4.48 (br, 1H), 3.46 (dd, *J*_1_ = 5.5 Hz, *J*_2_ = 16.2 Hz, 1H), 3.40 (dd, *J*_1_ = 7.3 Hz, *J*_2_ = 16.2 Hz, 1H), 2.32 (s, 3H) ppm; ^13^C NMR (100 MHz,CDCl_3_): δ 197.3, 147.1, 140.0, 139.9, 137.2, 135.2, 129.8 (×2), 129.7 (×2), 129.3 (×2), 129.1 (×2), 126.4 (×2), 118.0, 114.0 (×2), 54.6, 46.4, 21.2 ppm; IR (KBr) *ν*_max_ 3383, 1672, 1604, 1587, 1511, 1438, 1400, 1370, 1315, 1288, 1219, 1180, 1097, 1070, 995, 846, 815, 746 cm^−1^; HRMS (ESI+): *m*/*z* calcd for C_22_H_21_ClNO [M + H]^+^ 350.1306; found 350.1310.

*3-((4-Chlorophenyl)amino)-1,3-di-p-tolylpropan-1-one* (**16**): a white solid; mp 157–158 °C. ^1^H NMR (400 MHz, CDCl_3_): δ 7.78 (d, *J* = 8.2 Hz, 2H), 7.27 (d, *J* = 8.0 Hz, 2H), 7.21 (d, *J* = 8.0 Hz, 2H), 7.11 (d, *J* = 7.9 Hz, 2H), 7.00 (d, *J* = 8.8 Hz, 2H), 6.44 (d, *J* = 8.8 Hz, 2H), 4.89 (dd, *J*_1_ = *J*_2_ = 7.7 Hz, 1H), 3.42 (dd, *J*_1_ = 4.9 Hz, *J*_2_ = 16.2 Hz, 1H), 3.32 (dd, *J*_1_ = 7.9 Hz, *J*_2_ = 16.2 Hz, 1H), 2.38 (s, 3H), 2.30 (s, 3H) ppm; ^13^C NMR (100 MHz, CDCl_3_): δ 198.0, 145.9, 144.5, 139.7, 137.2, 134.4, 129.7 (×2), 129.5 (×2), 129.0 (×2), 128.5 (×2), 126.3 (×2), 122.4, 115.1 (×2), 54.9, 46.3, 21.8, 21.2 ppm; IR (KBr) *ν*_max_ 3396, 1666, 1605, 1511, 1490, 1404, 1367, 1317, 1292, 1178, 1109, 1087, 1002, 810, 730 cm^−1^; HRMS (ESI+): *m*/*z* calcd for C_23_H_23_ClNO [M + H]^+^ 364.8925; found 364.8927.

*1-(Phenylamino)-1-(o-tolyl)hexan-3-one* (**20**): colorless oil. ^1^H NMR (400 MHz, CDCl_3_): δ 7.40 ((t, *J* = 4.4 Hz, 1H), 7.20–7.17 (m, 3H), 7.13 (dd, *J*_1_ = *J*_2_ = 7.8 Hz, 2H), 6.70 (t, *J* = 7.3 Hz, 1H), 6.53 (d, *J* = 8.0 Hz, 2H), 5.08 (dd, *J*_1_ = 5.6 Hz, *J*_2_ = 7.4 Hz, 1H), 4.44 (br, 1H), 2.89 (dd, *J*_1_ = 5.3 Hz, *J*_2_ = 15.6 Hz, 1H), 2.83 (dd, *J*_1_ = 9.1 Hz, *J*_2_ = 17.0 Hz, 1H), 2.51 (s, 3H), 2.45–2.30 (m, 2H), 1.63–1.54 (m, 2H), 0.89 (t, *J* = 7.4 Hz, 3H) ppm; ^13^C NMR (100 MHz, CDCl_3_): δ 209.6, 147.0, 140.4, 134.9, 130.9, 129.3 (×2), 127.3, 126.7, 125.4, 117.8, 113.6 (×2), 51.0, 48.6, 45.5, 19.2, 17.0, 13.7 ppm; IR (KBr) *ν*_max_ 3402, 1671, 1602, 1519, 1448, 1434, 1406, 1363, 1344, 1318, 1287, 1258, 1220, 1201, 1182, 1099, 1016, 984, 853, 747 cm^−1^; HRMS (ESI+): *m*/*z* calcd for C_19_H_26_NO [M + H]^+^ 282.1852; found 282.1857.

*1-(2-Methoxyphenyl)-1-(phenylamino)hexan-3-one* (**21**): colorless oil. ^1^H NMR (400 MHz, CDCl_3_): δ 7.31 (d, *J* = 7.4 Hz, 2H), 7.24 (t, *J* = 7.8 Hz, 1H), 7.10(dd, *J*_1_ = *J*_2_ = 7.6 Hz, 2H), 6.92–6.86 (m, 2H), 6.65 (t, *J* = 7.3 Hz, 1H), 6.56 (d, *J* = 8.0 Hz, 2H), 5.15 (dd, *J*_1_ = *J*_2_ = 6.1 Hz, 1H), 4.73 (br, 1H), 3.93 (s, 3H), 2.97 (dd, *J*_1_ = 4.8 Hz, *J*_2_ = 15.2 Hz, 1H), 2.85(dd, *J*_1_ = 7.8 Hz, *J*_2_ = 15.2Hz, 1H), 2.42–2.32 (m, 2H), 1.59–1.50 (m, 2H), 0.85 (t, *J* = 7.4 Hz, 3H); ^13^C NMR (100 MHz, CDCl_3_): δ 210.4, 156.8, 147.2, 129.9, 129.2 (×2), 128.4, 127.8, 121.0, 117.7, 113.8 (×2), 110.7, 55.5, 50.3, 48.2, 45.3, 17.0, 13.8. ppm; IR (KBr) *ν*_max_ 3404, 3051, 1673, 1661, 1601, 1579, 1515, 1447, 1408, 1366, 1290, 1262, 1220, 1184, 1159, 1106, 1016, 984, 967, 917, 854, 789, 769, 747 cm^−1^; HRMS (ESI+): *m*/*z* calcd for C_19_H_25_NO_2_ [M + H]^+^ 298.1802; found 298.1803.

*1-((4-Chlorophenyl)amino)-1-(p-tolyl)hexan-3-one* (**22**): a white solid; mp 75–76 °C. ^1^H NMR (400 MHz, CDCl_3_) δ 7.21 (d, *J* = 8.0 Hz, 2H), 7.12 (d, *J* = 7.9 Hz, 2H), 7.02 (d, *J* = 8.8 Hz, 2H), 6.46 (d, *J* = 8.8 Hz, 2H), 4.75 (dd, *J*_1_ = *J*_2_ = 6.3 Hz, 1H), 4.59 (br, 1H), 2.87 (d, *J* = 6.4 Hz, 2H), 2.33–2.28 (m, 5H), 1.58–1.48 (m, 2H), 0.84 (t, *J* = 7.4 Hz, 3H) ppm; ^13^C NMR (100 MHz, CDCl_3_): δ 209.6, 145.7, 139.3, 137.3, 129.7 (×2), 129.1 (×2), 126.3 (×2), 122.5, 115.1 (×2), 54.6, 50.3, 45.8, 21.2, 17.0, 13.7 ppm; IR (KBr) *ν*_max_ 3383, 3029, 2965, 2930, 2876, 1711, 1602, 1510, 1487, 1453, 1407, 1376, 1354, 1318, 1286, 1247, 1177, 1020, 1084, 1051, 1019, 936, 814, 802, 775, 725 cm^−1^; HRMS (ESI+): *m*/*z* calcd for C_19_H_23_ClNO [M + H]^+^ 316.1463; found 316.1464.

*1-Phenyl-1-(m-tolylamino)hexan-3-one* (**23**): a white solid; mp 84–85 °C. ^1^H NMR (400 MHz, CDCl_3_): δ 7.37 (d, *J* = 7.2 Hz, 2H), 7.32 (dd, *J*_1_ = *J*_2_ = 7.4 Hz, 2H), 7.25 (d, *J* = 8.6 Hz, 1H), 6.99 (t, *J* = 7.8 Hz, 1H), 6.50 (d, *J* = 7.4 Hz, 1H), 6.42 (s, 1H), 6.35 (d, *J* = 8.0 Hz, 1H), 4.85 (dd, *J*_1_ = *J*_2_ = 6.4 Hz, 1H), 4.47 (br, 1H), 2.89 (d, *J* = 6.4 Hz, 2H), 2.34–2.29 (m, 2H), 2.22 (s, 3H), 1.58–1.49 (m, 2H), 0.84 (t, *J* = 7.4 Hz, 3H) ppm; ^13^C NMR (100 MHz, CDCl_3_): δ 209.6, 147.1, 142.9, 139.0, 129.2, 128.9 (×2), 127.4, 126.5 (×2), 118.9, 114.8, 110.9, 54.7, 50.4, 45.8, 21.7, 17.1, 13.7 ppm; IR (KBr) *ν*_max_ 3377, 3027, 2960, 2932, 2876, 1709, 1606, 1594, 1525, 1492, 1483, 1454, 1408, 1333, 1307, 1287, 1253, 1182, 1165,1119, 1091, 1055, 861, 848, 778 cm^−1^; HRMS (ESI+): *m*/*z* calcd for C_19_H_25_NO [M + H]^+^ 282.1852; found 282.1853.

*4-Methyl-1-(phenylamino)-1-(p-tolyl)pentan-3-one* (**25**): a white solid; mp 77–78 °C. ^1^H NMR (400 MHz, CDCl_3_): δ 7.39 (d, *J* = 7.8 Hz, 2H), 7.26–7.21 (m, 4H), 6.79 (t, *J* = 7.3 Hz, 1H), 6.69 (d, *J* = 8.1 Hz, 2H), 4.95 (dd, *J*_1_ = *J*_2_ = 6.2 Hz, 1H), 4.71 (br, 1H), 3.07 (d, *J* = 6.3 Hz, 2H), 2.66–2.56 (m, 1H), 2.44 (s, 3H), 1.13 (t, *J* = 6.2 Hz, 6H) ppm; ^13^C NMR (100 MHz, CDCl_3_): δ 213.2, 147.2, 139.9, 137.0, 129.6 (×2), 129.2 (×2), 126.4 (×2), 117.8, 113.9 (×2), 54.4, 48.1, 41.7, 21.2, 17.9, 17.8 ppm; IR (KBr) *ν*_max_ 3376, 3051, 3025, 2969, 2931, 1705, 1604, 1514, 1496, 1464, 1439, 1419, 1381, 1364, 1317, 1284, 1180, 1104, 1076, 1021, 867, 817, 749, 731 cm^−1^; HRMS (ESI+): *m*/*z* calcd for C_19_H_24_NO [M + H]^+^ 282.1852; found 282.1855.

*1-((4-Chlorophenyl)amino)-4-methyl-1-(o-tolyl)pentan-3-one* (**27**): a white solid; mp 77–78 °C. ^1^H NMR (400 MHz, CDCl_3_): δ 7.32 (t, *J* = 4.8 Hz, 1H), 7.19–7.13 (m, 3H), 7.02 (d, *J* = 8.7 Hz, 2H), 6.39 (d, *J* = 8.7 Hz, 2H), 4.97 (dd, *J*_1_ = *J*_2_ = 6.7 Hz, 1H), 4.61 (br, 1H), 2.91(dd, *J*_1_ = 5.0 Hz, *J*_2_ = 16.1 Hz, 1H), 2.85 (dd, *J*_1_ = 7.8 Hz, *J*_2_ = 16.4 Hz,, 2.53–2.46 (m, 1H), 2.45 (s, 3H), 1.02 (dd, *J*_1_ = 3.8Hz, *J*_2_ = 6.8 Hz, 6H) ppm; ^13^C NMR (100 MHz, CDCl_3_): δ 213.2, 145.7, 140.2, 134.9, 131.1, 129.1 (×2), 127.5, 126.8, 125.4, 122.5, 114.8 (×2), 51.2, 46.0, 41.7, 19.3, 17.9 ppm. IR (KBr) *ν*_max_ 3393, 3055, 3031, 1669, 1604, 1578, 1511, 1489, 1448, 1437, 1402, 1370, 1317, 1287, 1218, 1183, 1094, 1070, 1015, 1002, 991 cm^−1^; HRMS (ESI+): *m*/*z* calcd for C_19_H_24_ClNO [M + H]^+^ 316.1463; found 316.1464.

*1-(2-Methoxyphenyl)-4-methyl-1-(m-tolylamino)pentan-3-one* (**28**): a white solid; mp 169–170 °C. ^1^H NMR (400 MHz, CDCl_3_): δ 7.32 (d, *J* = 7.4 Hz, 1H), 7.22 (t, *J* = 7.2 Hz, 1H), 6.99 (t, *J* = 7.8 Hz, 1H), 6.91–6.86 (m, 2H), 6.49 (d, *J* = 7.4 Hz, 1H), 6.44 (s, 1H), 6.36 (d, *J* = 7.9 Hz, 1H), 5.14 (dd, *J*_1_ = *J*_2_ = 6.1 Hz, 1H), 4.79 (br, 1H), 3.94 (s, 3H), 3.05 (dd, *J*_1_ =5.0 Hz, *J*_2_ *=* 15.5 Hz, 1H), 2.90 (dd, *J*_1_ = 7,5 Hz, *J*_2_ = 15.5 Hz, 1H), 2.59–2.48 (m, 1H), 2.23 (s, 3H), 1.02 (dd, *J*_1_ = 3.0 Hz, *J*_2_ = 7.0 Hz, 6H) ppm; ^13^C NMR (100 MHz, CDCl_3_): δ 213.8, 156.8, 147.2, 138.8, 130.2, 129.1, 128.2, 127.9, 121.0, 118.6, 114.8, 110.8, 110.6, 55.4, 50.5, 45.7, 41.3, 21.7, 17.9, 17.7 ppm. IR (KBr) *ν*_max_ 3379, 3063, 3024, 1672, 1604, 1587, 1567, 1509, 1492, 1454, 1437, 1401, 1371, 1352, 1289, 1218, 1181, 1098, 1080, 996, 848, 821, 793, 759, 746 cm^−1^; HRMS (ESI+): *m*/*z* calcd for C_20_H_28_NO_2_ [M + H]^+^ 312.1958; found 312.1960.

*1-((4-Chlorophenyl)amino)-4-methyl-1-(p-tolyl)pentan-3-one* (**29**): a white solid; mp 91–92 °C. ^1^H NMR (400 MHz, CDCl_3_): δ 7.22 (d, *J* = 7.7 Hz, 2H), 7.12 (d, *J* = 7.6 Hz, 2H), 7.03 (d, *J* = 8.2 Hz, 2H), 6.47 (d, *J* = 8.3 Hz, 2H), 4.76 (m, 1H), 4.69 (s, 1H), 2.93 (d, *J* = 6.0 Hz, 2H), 2.51–2.44 (m, 1H), 2.32 (s, 3H), 1.00 (d, *J* = 6.9 Hz, 6H) ppm; ^13^C NMR (100 MHz, CDCl_3_): δ 213.2, 145.8, 139.4, 137.2, 129.6 (×2), 129.1 (×2), 126.3 (×2), 122.4, 115.0 (×2), 54.6, 47.9, 41.8, 21.2, 17.8 (×2) ppm; IR (KBr) *ν*_max_ 3369, 3028, 2965, 2933, 2870, 1703, 1605, 1508, 1489, 1464, 1423, 1380, 1361, 1314, 1281, 1246, 1177, 1110, 1089, 1070, 1022, 819, 808, 731 cm^−1^; HRMS (ESI+): *m*/*z* calcd for C_19_H_23_ClNO [M + H]^+^ 316.1463; found 316.1461.

## 4. Conclusions

In summary, Hf(OTf)_4_ was identified as a highly efficient catalyst for Mannich reaction. Under solvent-free conditions, as low as 0.1–0.5 mol% Hf(OTf)_4_ could catalyze high-yielding formation of a diversity of aryl and alkyl ketone-based Mannich bases. The presence of Hf(OTf)_4_ resulted in excellent region- and diastereoselectivity in the synthesis of alkyl ketone-based Mannich bases. ^1^H NMR tracing of the H/D exchange reactions of acetophenone and cyclopentanone in MeOH-*d*_4_ illustrated that the coordination of Hf(OTf)_4_ with ketone could enable its fast keto-enol tautomerization, thereby contributing to the overall promotion of Mannich reaction.

## Figures and Tables

**Table 1:**
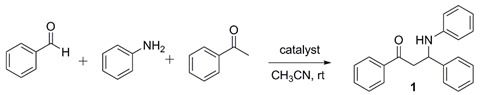
The catalytic effect of Group IVB transition metal Lewis acids on Mannich reaction ^a^.

	Catalyst (5 mol%)	Reaction Time (h)	Yield of 1 (%)
1	no	24	no reaction
2	ZrCl_4_	24	70
3	ZrOCl_2_·8H_2_O	24	71
4	ZrCp_2_Cl_2_	12	73
5	HfCl_4_	8	81
6	Hf(OTf)_4_	6	89

^a^ Benzaldehyde/aniline/acetophenone are in a 1:1:2 molar ratio.

**Table 2:**
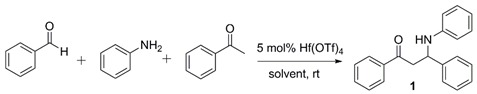
Solvent effect on the Hf(OTf)_4_-catalyzed Mannich reaction ^a^.

	Solvent	Reaction Time (h)	Yield of 1 (%)
1	THF	36	82
2	DME	36	80
3	benzene	24	73
4	CH_2_Cl_2_	24	68
5	CH_3_CN	6	89
6	EtOH	5	94

^a^ Benzaldehyde/aniline/acetophenone are in a 1:1:2 molar ratio.

**Table 3:**
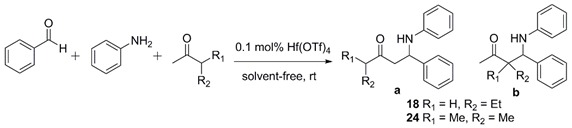
The regioselectivity of Hf(OTf)_4_-catalyzed synthesis of Mannich bases **18** and **24** ^a^.

	Compd	Hf(OTf)_4_ (mol%)	Reaction Time (h)	Yield (%)
1	**18**	-	48	71 (**a**:**b** = 1:0.53)
2	**18**	0.1	4	89 (**a** only)
3	**24**	-	72	15 (**a**:**b** = 1:0.48)
4	**24**	0.1	6	87 (**a** only)

^a^ Benzaldehyde/aniline/alkyl ketone are in a 1:1:2 molar ratio.

**Table 4:**
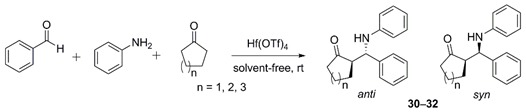
The diastereoselectivity of Hf(OTf)_4_-catalyzed synthesis of cycloketone-derived Mannich bases **30**–**32** ^a^.

	Compd	n	Hf(OTf)_4_ (mol%)	Reaction Time (h)	Yield (%)	*anti*/*syn*
1	**30**	1	-	12	-	-
2	**30**	1	0.1	0.5	89	92:8
3	**31**	2	-	6	71	63:37
4	**31**	2	0.1	0.33	92	96:4
5	**32**	3	-	48	58	20:80
6	**32**	3	0.1	8	88	59:41
7	**32**	3	1	1	90	68:32
8	**32**	3	10	0.16	89	77:23
9	**32**	3	50	0.05	82	86:14

^a^ Aldehyde/aniline/cycloketone are in a 1:1:2 molar ratio.

## Supplementary Materials

The following are available online, Figure S1–S22: The NMR spectra of new compounds **11**, **15**, **16**, **2****0**–**2****3**, **2****5**, and **27**–**29**.
