# Supplementary material for: Hf(OTf)4 as a Highly Potent Catalyst for the Synthesis of Mannich Bases under Solvent-Free Conditions
Source: Molecules. 2020 Jan 17;25(2):388. doi: 10.3390/molecules25020388 (PMC7024362; doi:10.3390/molecules25020388)
Supplement: Supplementary file 1 [file molecules-25-00388-s001.pdf]

<u>Supporting Information</u>

$Hf(OTf)_4$ as a Highly Potent Catalyst for the Synthesis of Mannich Bases under Solvent-Free Conditions

Shuai-Bo Han, Jing-Ying Wei, Xiao-Chong Peng, Rong Liu,
Shan-Shan Gong* and Qi Sun*

Jiangxi Key Laboratory of Organic Chemistry, Jiangxi Science and Technology Normal University, 605 Fenglin Avenue, Nanchang, Jiangxi 330013, PR China

E-mails: gongshanshan@jxstnu.edu.cn;sunqi@jxstnu.edu.cn

The NMR spectra of Mannich bases 11, 15, 16, 20–23, 25 and 27–29

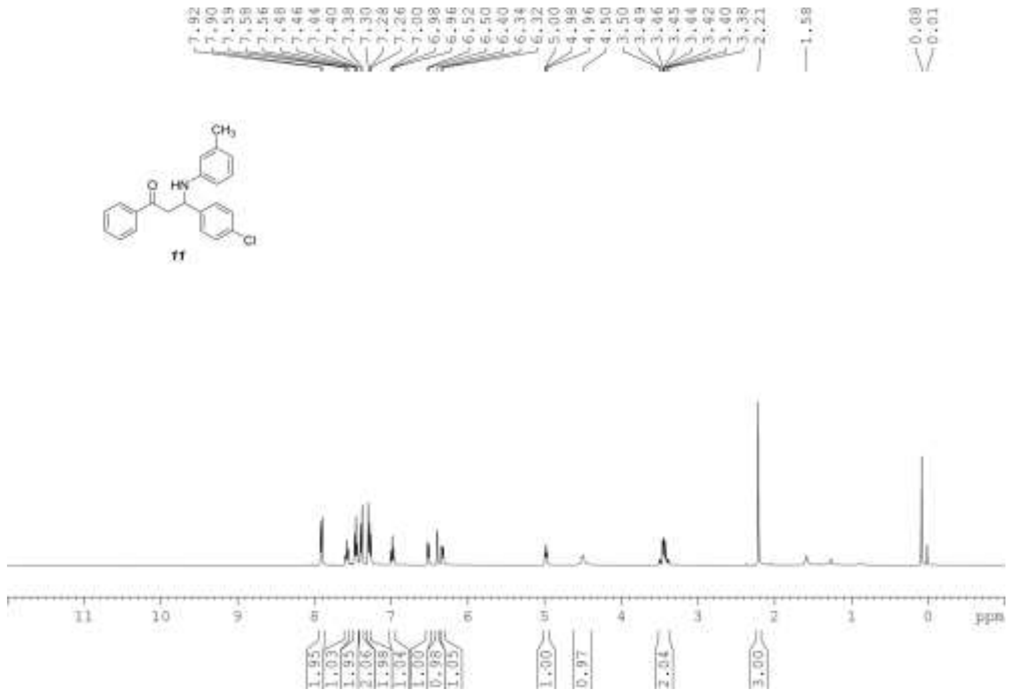

Figure S1. ^{1}H NMR of **11**

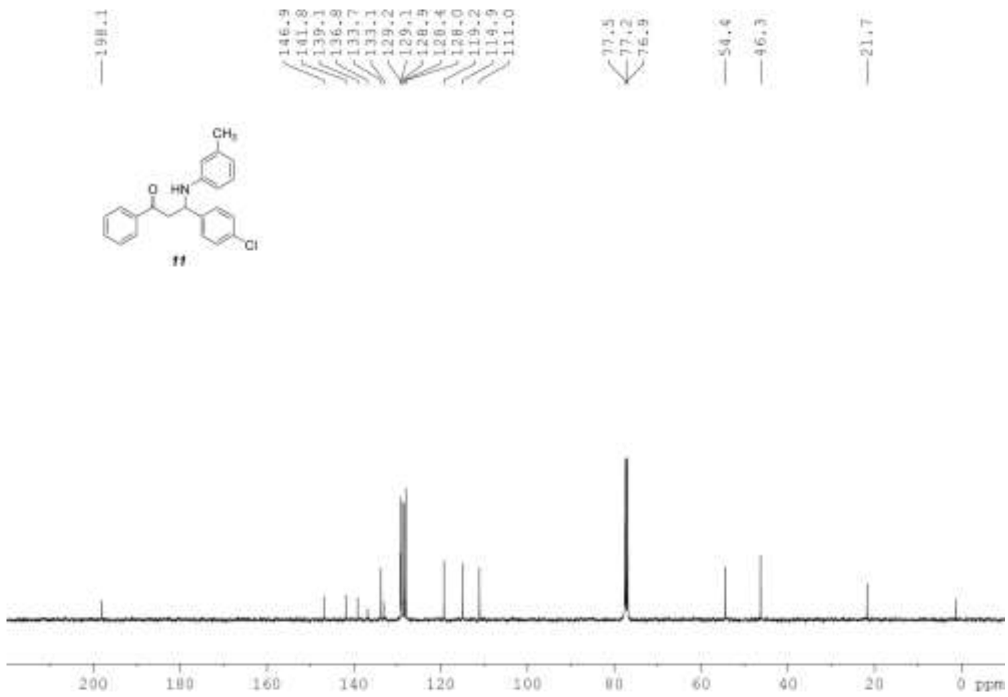

Figure S2. ^{13}C NMR of **11**

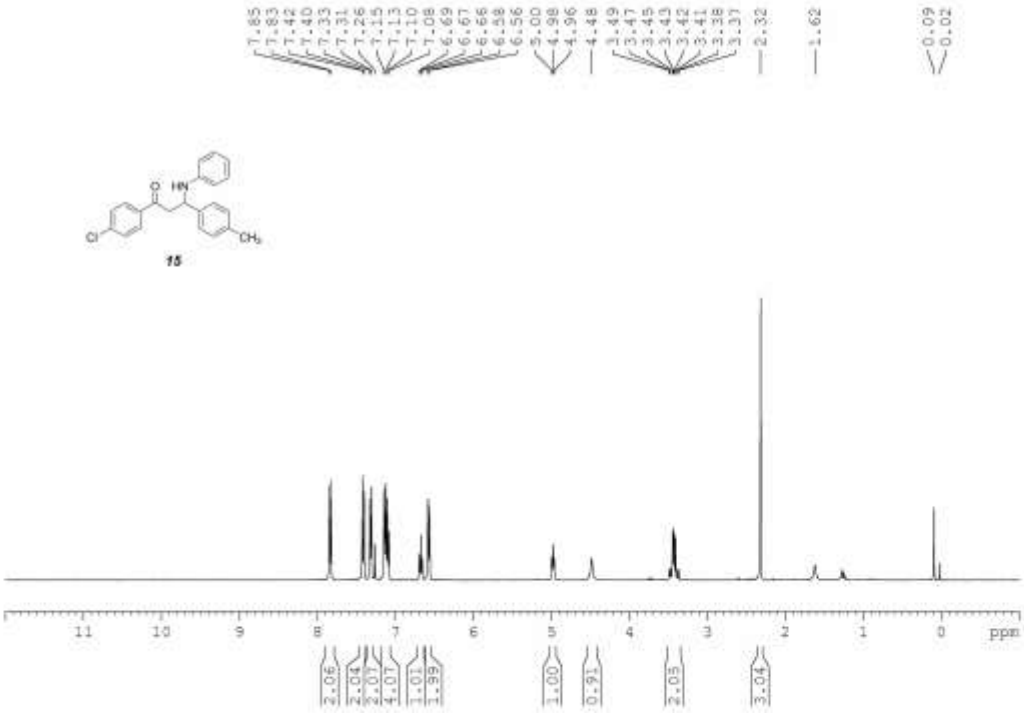

Figure S3. ^{1}H NMR of **15**

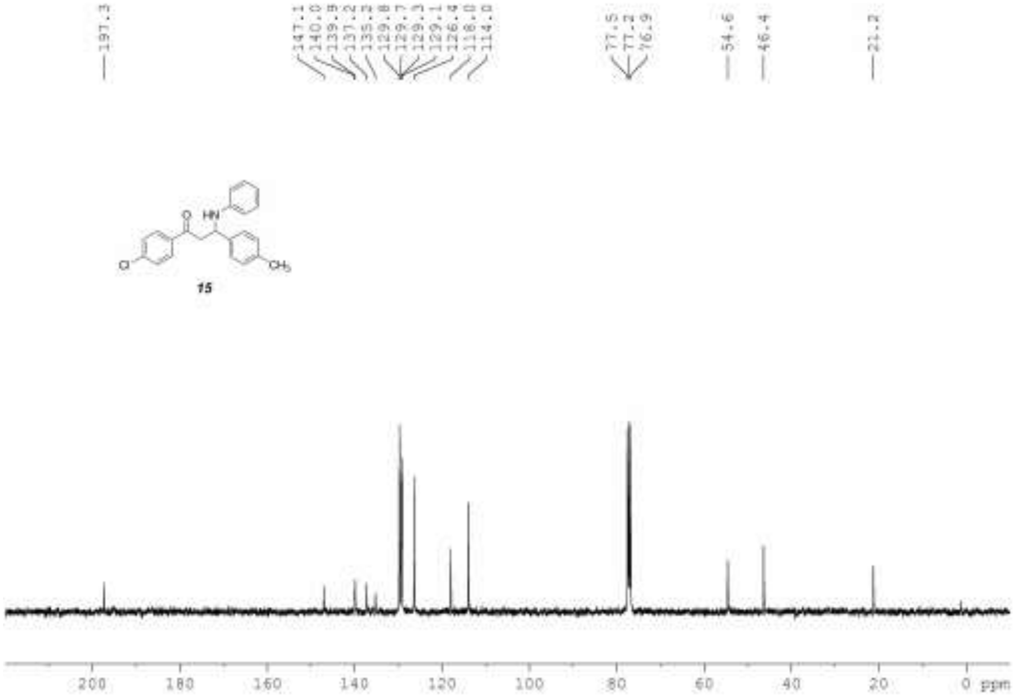

Figure S4. ^{13}C NMR of **15**

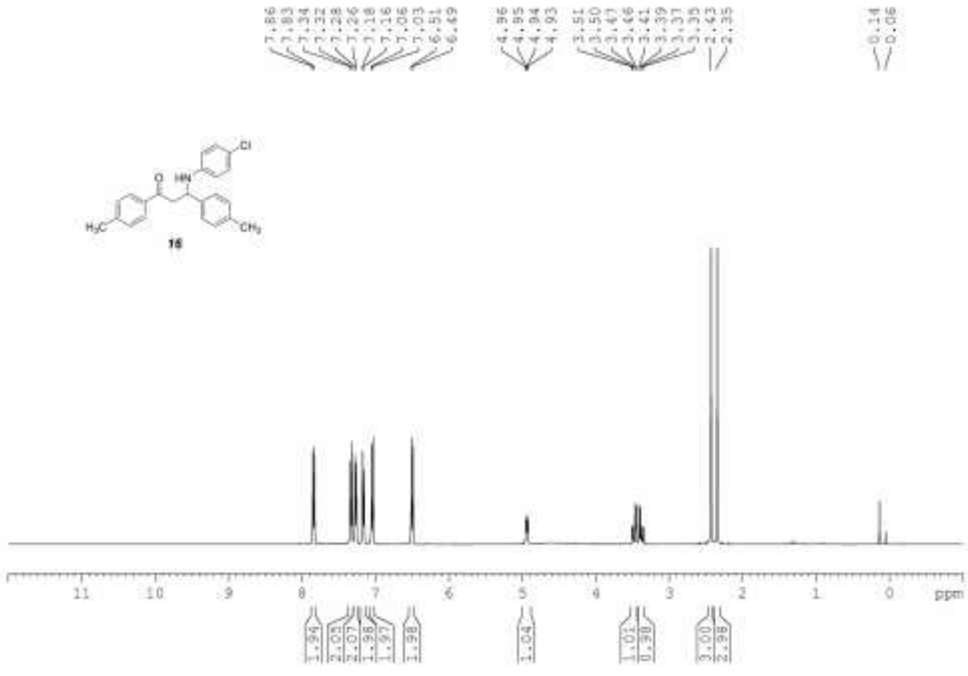

Figure S5. ^{1}H NMR of **16**

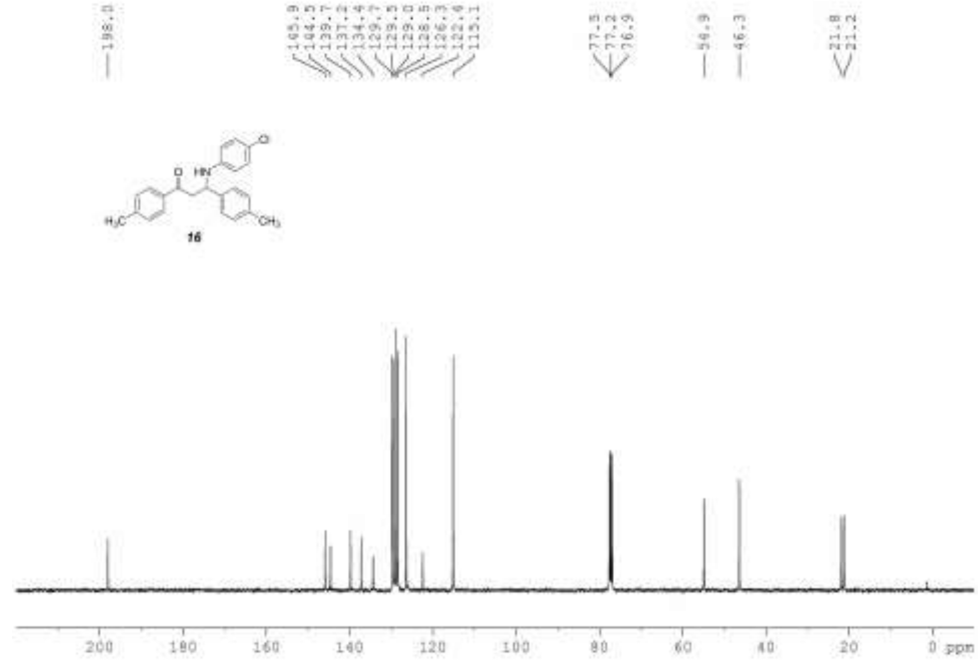

Figure S6. ^{13}C NMR of **16**

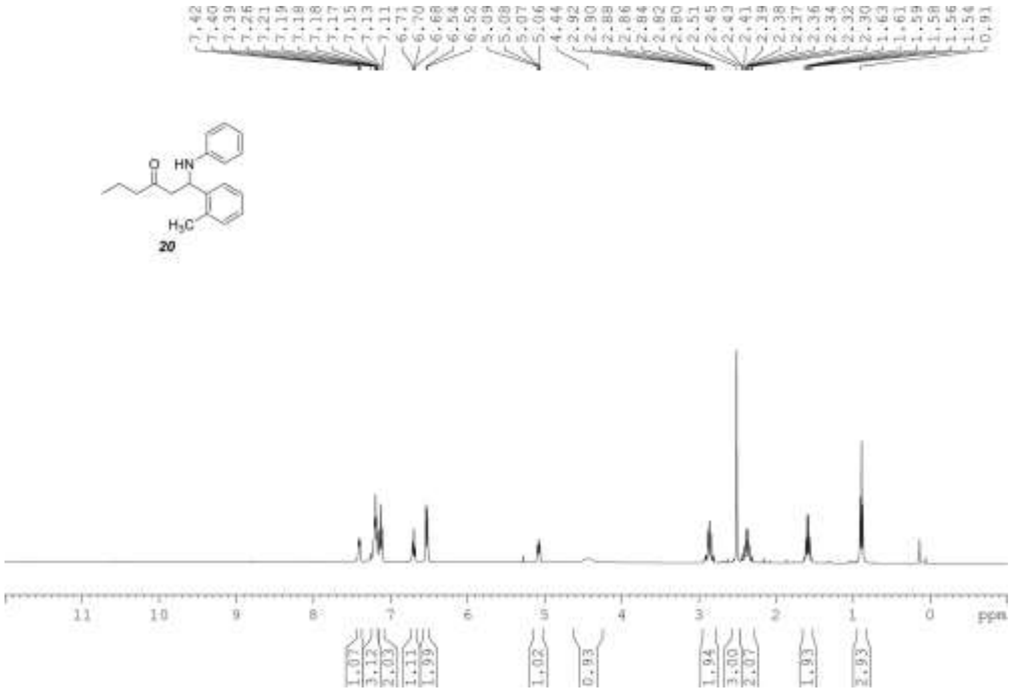

Figure S7. ^{1}H NMR of **20**

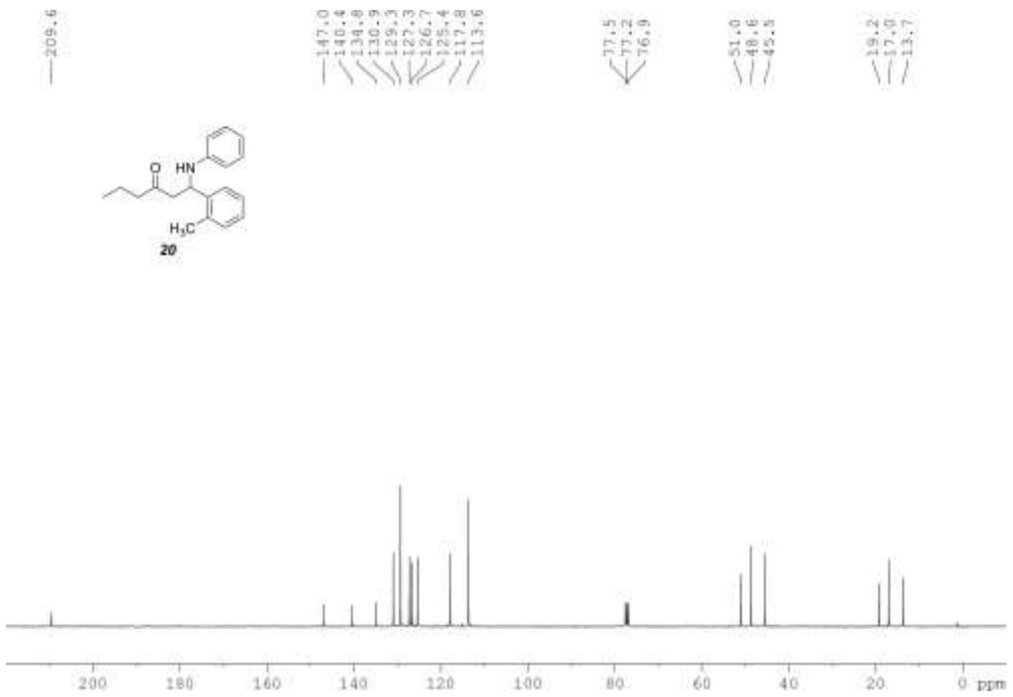

Figure S8. ^{13}C NMR of **20**

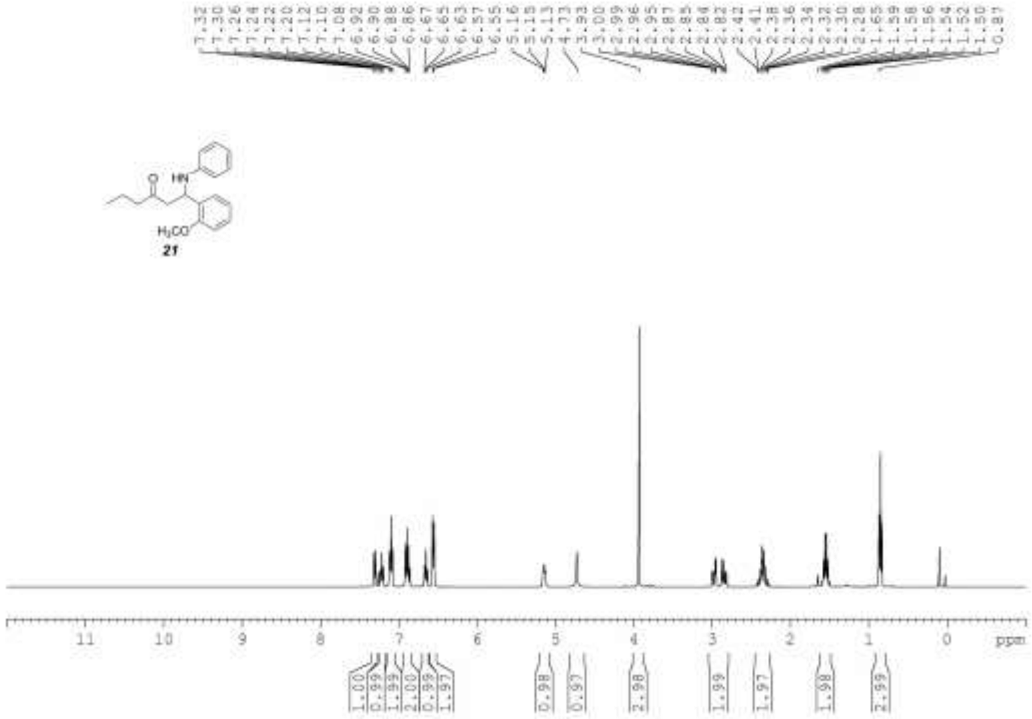

Figure S9. ^{1}H NMR of **21**

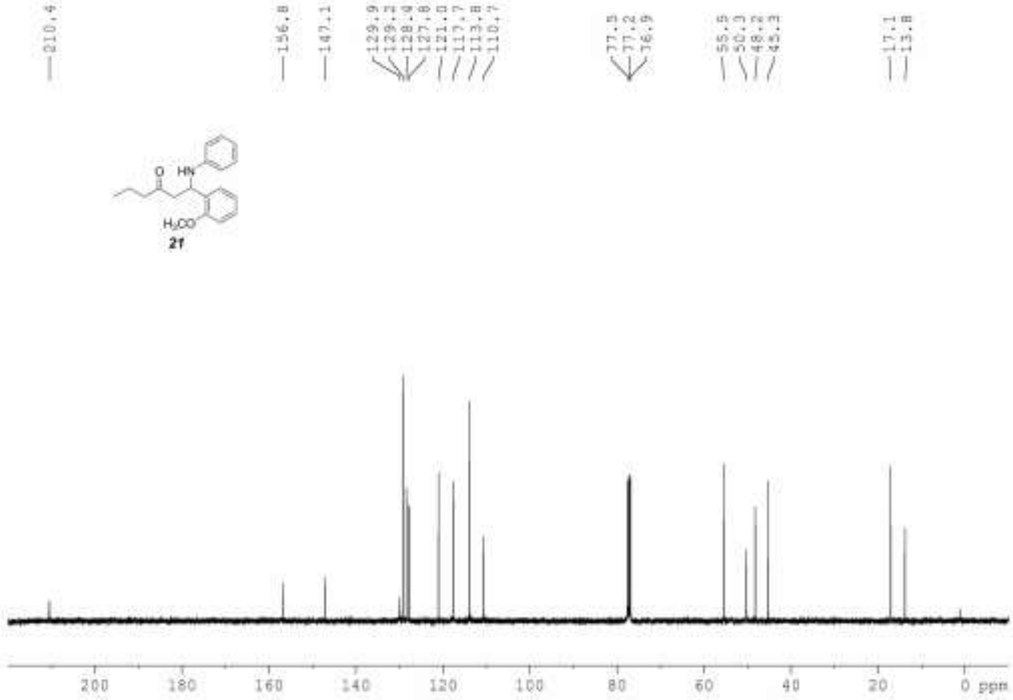

Figure S10. ^{13}C NMR of **21**

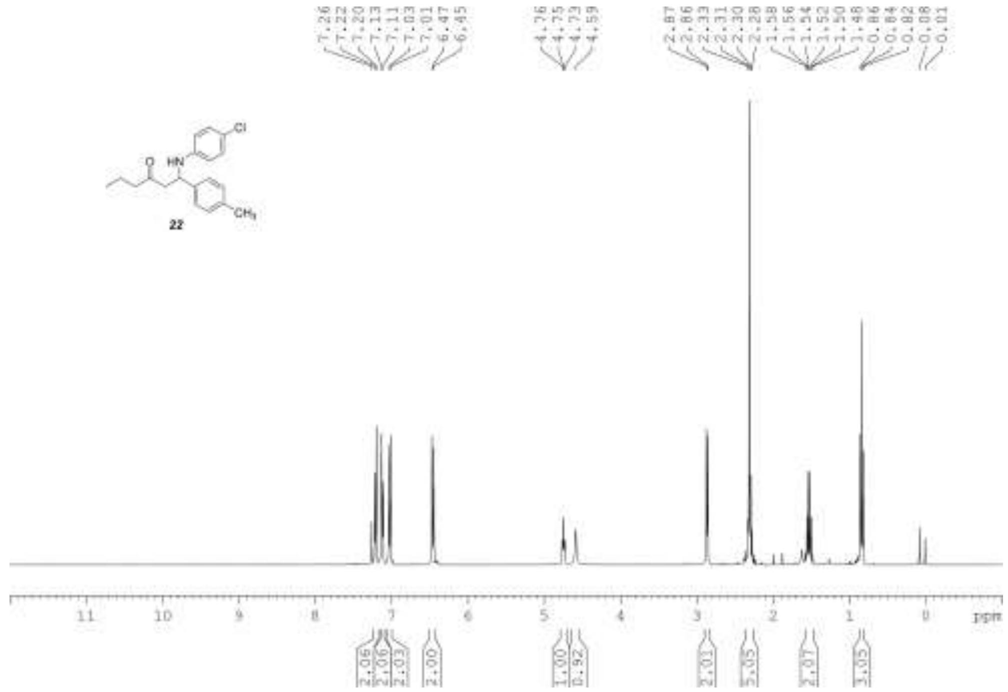

Figure S11. ^{1}H NMR of **22**

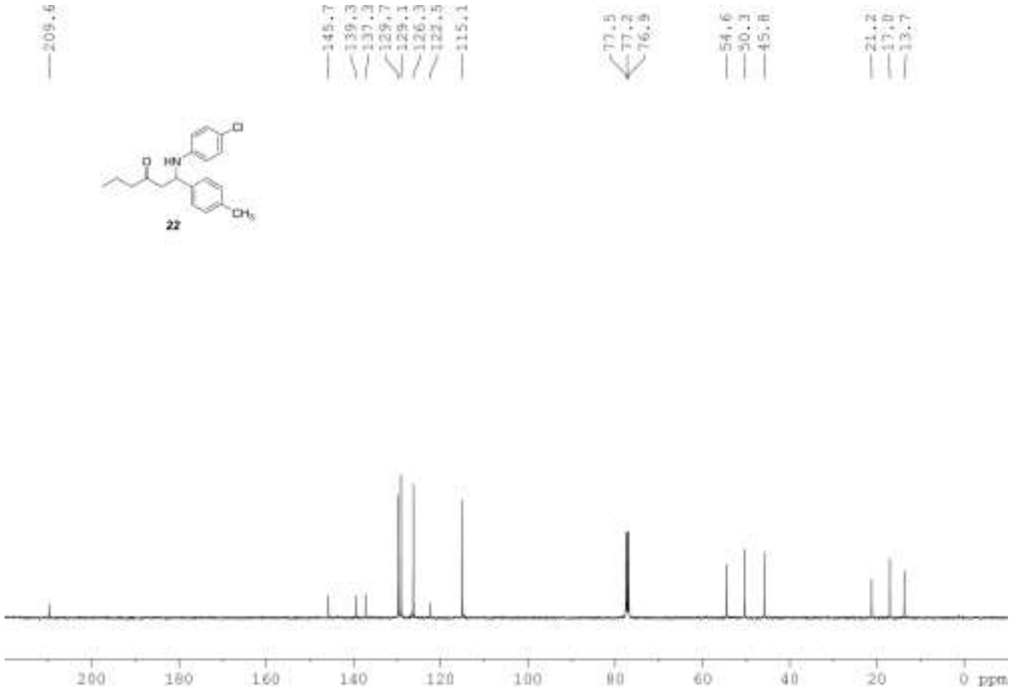

Figure S12. ^{13}C NMR of **22**

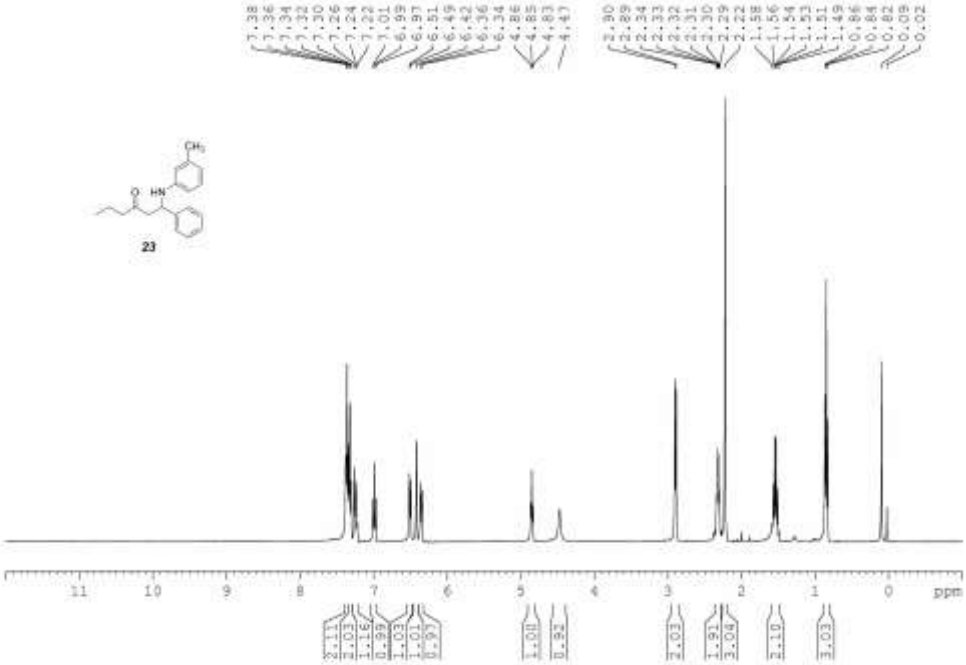

Figure S13. ^{1}H NMR of **23**

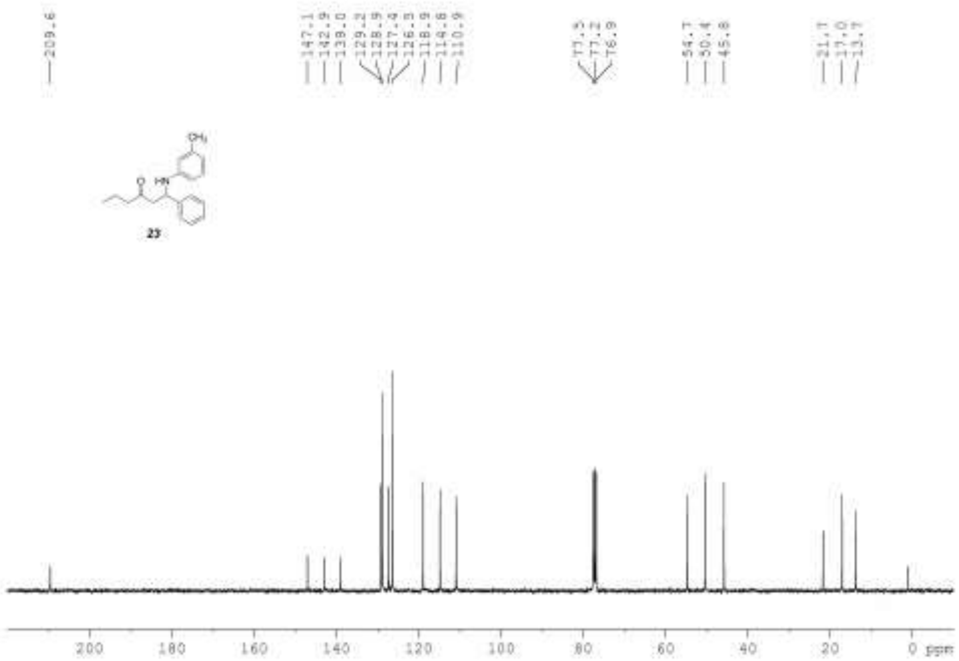

Figure S14. ^{13}C NMR of **23**

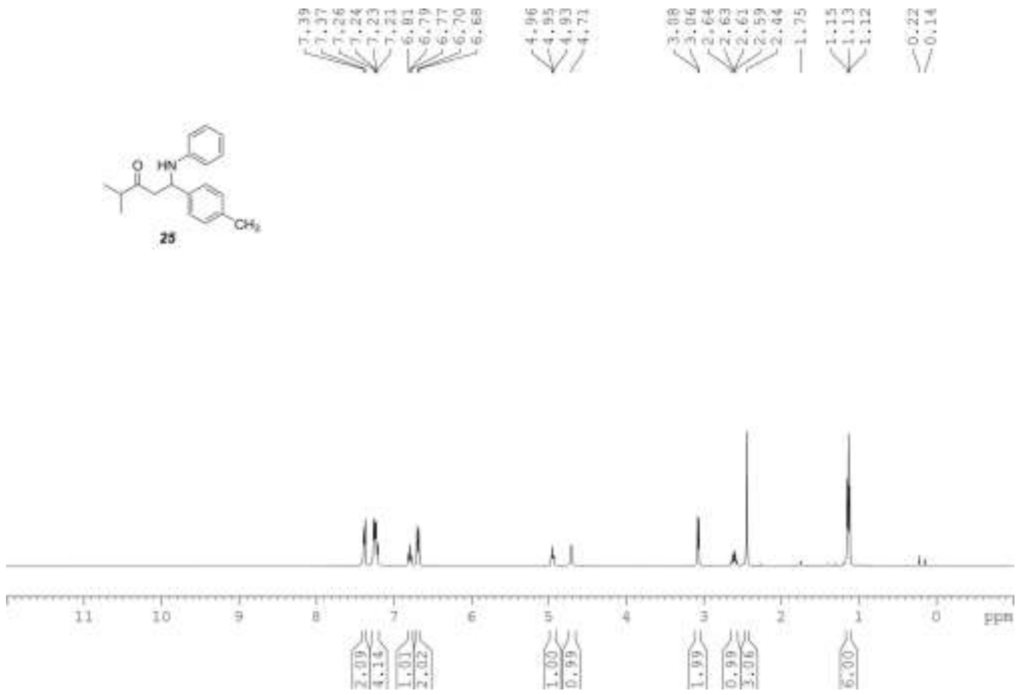

Figure S15. ^{1}H NMR of **25**

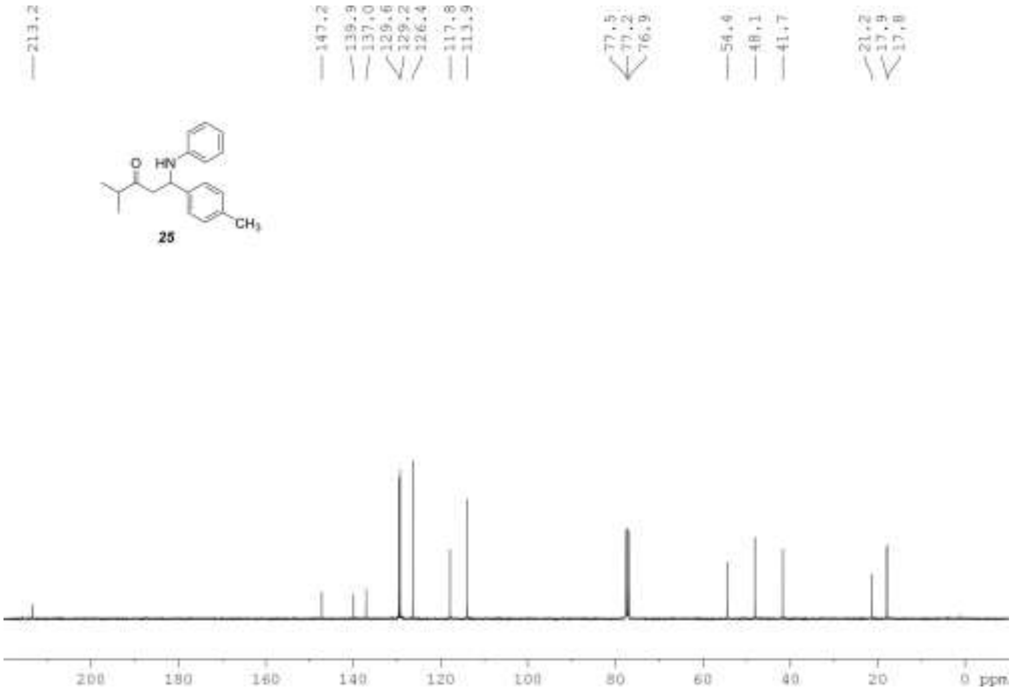

Figure S16. ^{13}C NMR of **25**

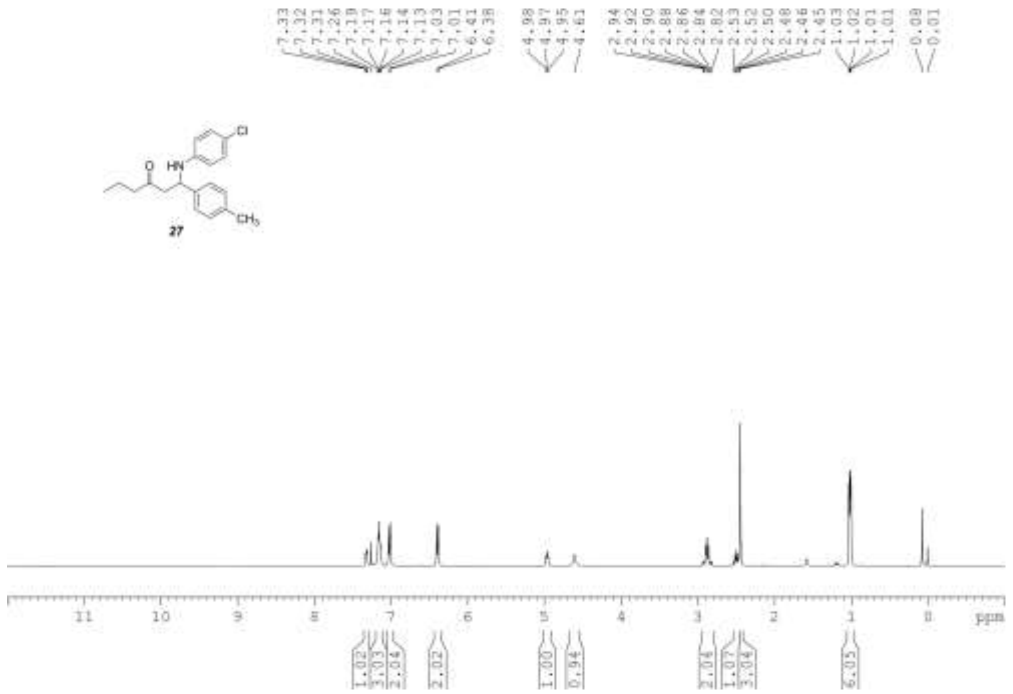

Figure S17. ^{1}H NMR of **27**

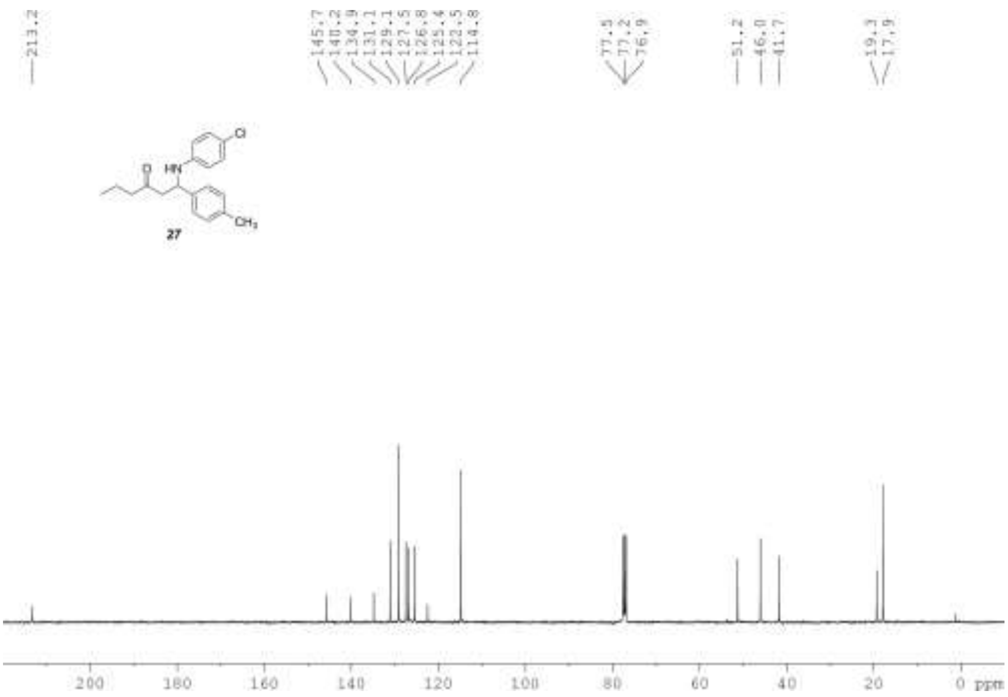

Figure S18. ^{13}C NMR of **27**

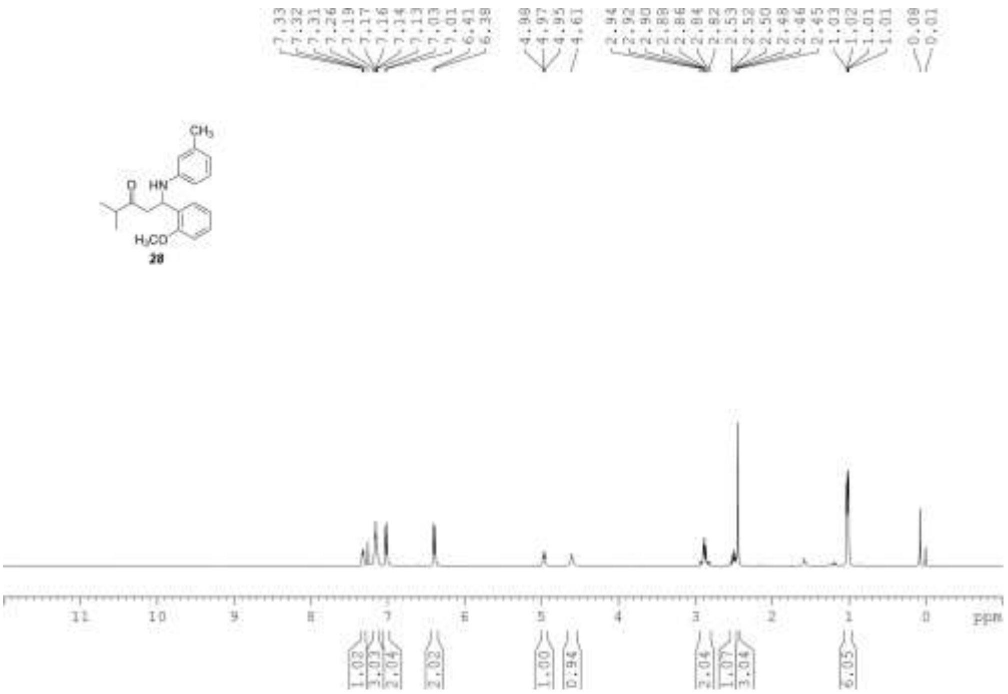

Figure S19. ^{1}H NMR of **28**

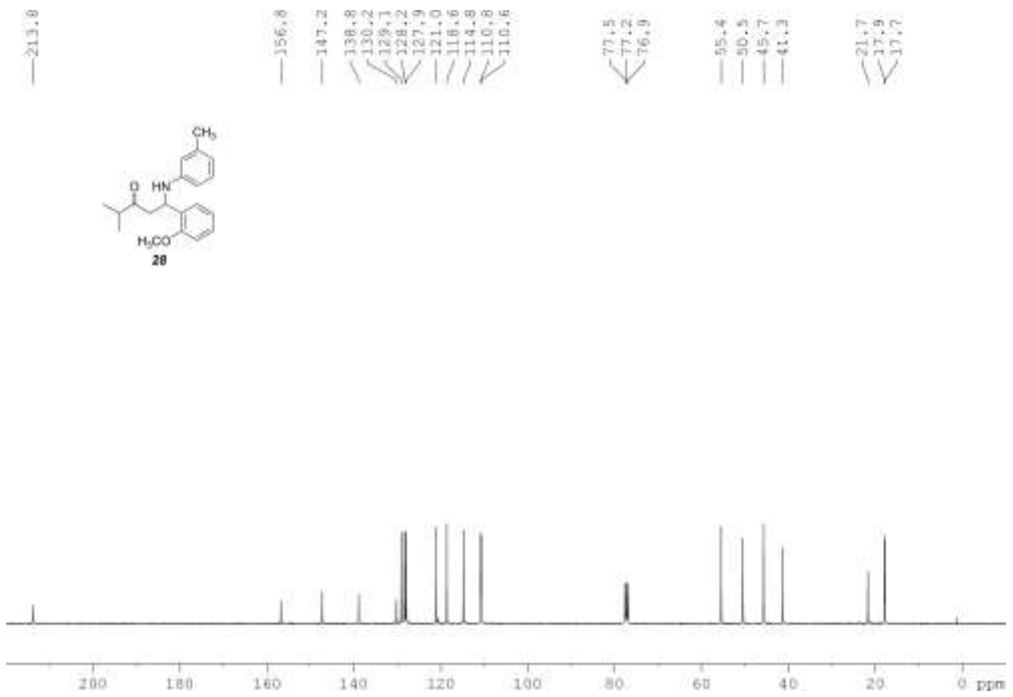

Figure S20. ^{13}C NMR of **28**

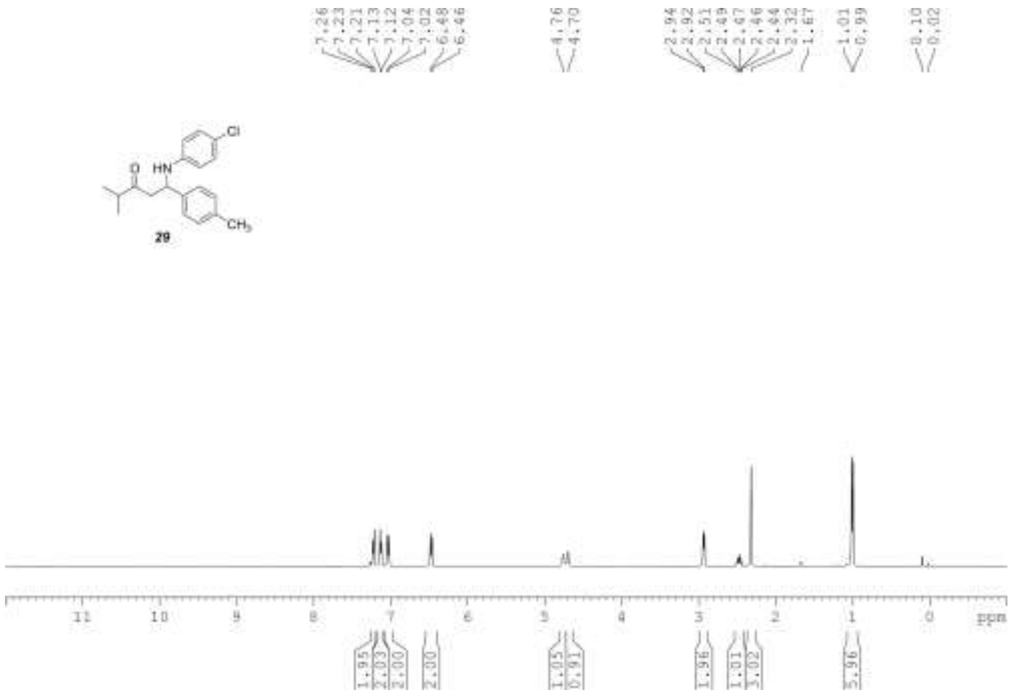

Figure S21. ^{1}H NMR of **29**

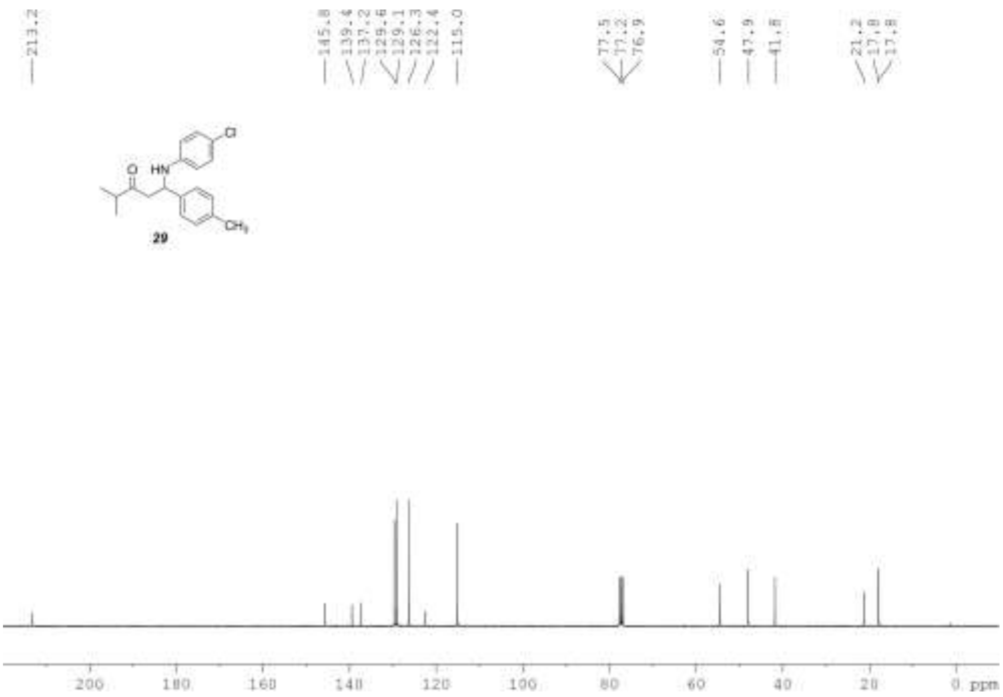

Figure S22. ^{13}C NMR of **29**
